# Near-fatal anaphylaxis with Kounis syndrome caused by *Argas reflexus* bite: a case report

**DOI:** 10.1186/s12948-020-00121-w

**Published:** 2020-03-18

**Authors:** Elisa Boni, Cristoforo Incorvaia

**Affiliations:** 1 Allergy Unit, Hospital Santo Spirito ASL AL, Via G. Giolitti 2, 15033 Casale Monferrato, Alessandria, Italy; 2 Cardiac/Pulmonary Rehabilitation Unit, ASST Pini-CTO, Milan, Italy

**Keywords:** Argas reflexus, Tick bites, Anaphylaxis, Kounis syndrome

## Abstract

**Background:**

The pigeon tick *Argas reflexus* is a temporary parasite of pigeons. It bites during night hours and lies briefly on its prey, as long as it takes the blood meal. When pigeons are not accessible, ticks look for other hosts, invading nearby flats and biting humans.

**Case presentation:**

We present the case of a woman aged 46 years who experienced severe anaphylaxis during the night which required emergency medical treatment, tracheal intubation and hospitalization in intensive care unit. Kounis syndrome was documented by transient ST depression and elevation of troponin. The allergological work up ruled out hypersensitivity to drugs, latex and foods containing alpha-gal, which is a cause of anaphylaxis. Basal serum tryptase was in normal range (8.63 ng/ml). When questioned about the presence of ticks, the patient brought into view various specimens of ticks that were recognized by an entomologist as *Argas reflexus*.

**Conclusions:**

An in vitro diagnosis of allergy to *Argas reflexus* is currently not feasible because, though the major allergen Arg r 1 has been isolated, allergen extracts are not commercially available. Therefore, the diagnosis of anaphylaxis from *Argas reflexus*, when other causes of anaphylaxis are excluded, must rely only on history and clinical findings, as well as on the presence of pigeons and/or pigeon ticks in the immediate domestic environment.

## Background

The pigeon tick *Argas reflexus* is a temporary parasite of pigeons (*Columba livia*) in Southern and Central Europe. It belongs to the Argasidae or soft tick family [1] and has been described for the first time in Italy. It is a nidicolous, endophilic, polyphasic and monotropic soft tick which originally parasitized several species of wild birds, whose favourite host is pigeons. As a consequence of pigeon domestication, *A. reflexus* colonized rural and urban environments [2]. It therefore lives where pigeons nests are common, such as old urban housing or higher floors, but it may also be present in old renovated places such as attics. *A. reflexus* bites during night hours and lies briefly on its prey, as long as it takes the blood meal. When pigeons are not within reach, ticks look for other preys invading nearby flats and bite humans.

Clinical manifestation induced by tick bites are local oedema and erythema but systemic reactions can occur. Anaphylaxis is defined as a serious, potentially life-threatening generalized hypersensitivity reaction with rapid onset. Clinical manifestation is usually characterized by involvement of at least two different organs (including skin, respiratory, cardiovascular or gastrointestinal systems) although isolated severe hypotension may be the only clinical feature in some patients. Usually a transient increase of tryptase of at least 20% above baseline plus 2 ng/ml is also detectable within 4 h of the reaction [3]. Acute coronary syndrome may occur during anaphylaxis either through vasospasm or through acute plaque rupture and thrombus formation. This condition is known as Kounis syndrome [4, 5]. Nocturnal anaphylaxis is rare. When it occurs, delayed anaphylaxis due to red meat allergy in patients sensitized to alpha-gal has to be suspected [6]. Bites from insects or ticks during night time also have to be considered. The dominant allergen Arg r 1 of 18 to 19 kd has been isolated in *A. reflexus.* Arg r 1 is a lipocalin and has been used as diagnostic in vitro and in vivo tool in a series of anaphylaxis caused by the pigeon soft tick [7, 8]. Lipocalins are a family of extracellular proteins with a molecular weight of about 20 kd with great structural and functional diversity. They include allergens from dog, cow, horse, cockroach; they show only about 20% amino acid sequence homology. Arg r 1 has a 25–35% sequence identity with known other tick lipocalin [7]. However, diagnosis of allergy to *A. reflexus* is hampered by the unavailability of commercial tests for routine use. In vivo tests with tick extracts are concerned by the risk of transferring infectious agents.

## Case presentation

**Fig. 1:**
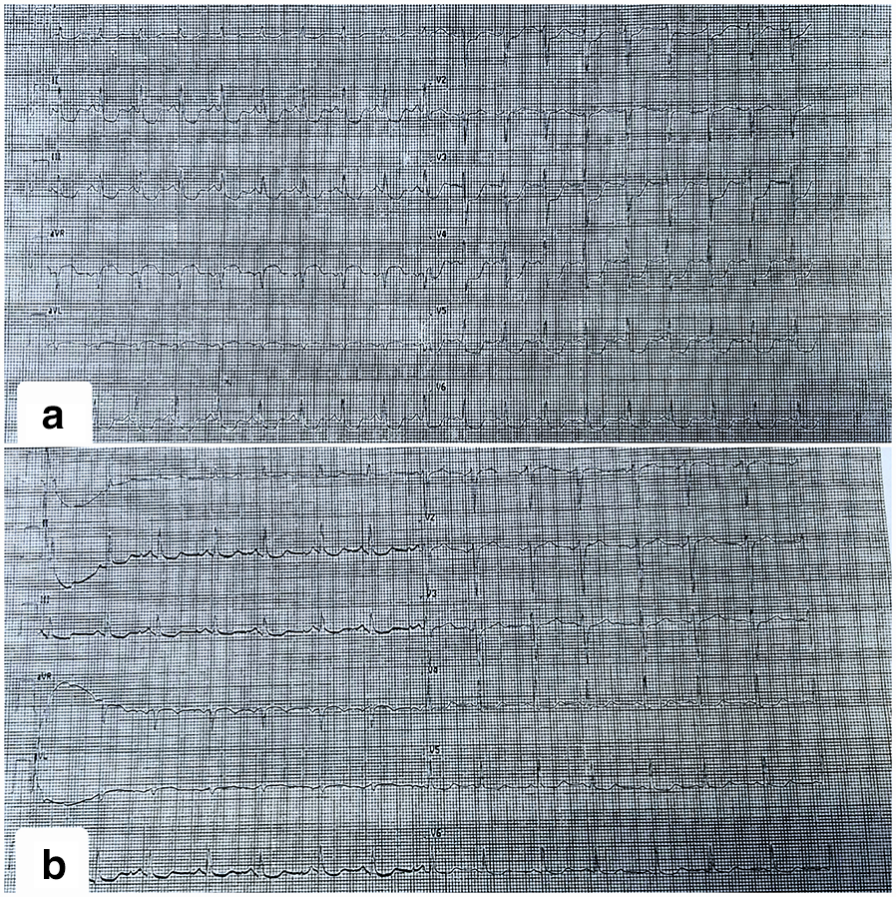
Electrocardiography. **a** ECG 90 min after the onset of symptoms with ST depression in antero-lateral and inferior leads and specular elevation in aVR. **b** ECG 3 hours after the onset of symptoms: normal exam

We present the case of a woman aged 46 years suffering from arterial hypertension in treatment with nebivolol and lisinopril. She experienced severe anaphylaxis which awakened her from sleep during the night and required emergency medical treatment, tracheal intubation and hospitalization in intensive care unit. The clinical presentation included generalized urticaria, angioedema of lips, hands and feet, dyspnoea and oxygen desaturation (SpO_2_ 49%), hypotension (blood pressure 70/30) and tachycardia (150 bpm), severe diarrhoea with hypoxemic acidosis and loss of consciousness. No acute serum tryptase measurement was performed in emergency room. Troponin elevation was observed (Table 1) and the electrocardiogram (ECG) showed a ST segment depression in antero-lateral and inferior leads and specular elevation in aVR, suggestive of myocardial ischemia. The ECG returned normal a few hours later as well as troponin levels. (Fig. 1). No abnormalities were detected in transthoracic cardiac echography. This condition defines the Kounis syndrome.

**Table 1:** Cardiac enzymes values

Measurement sequence	Troponin (ng/l)
0	0.03
1	0.10
2	0.26
3	0.23
4	0.13
5	0.05

**Fig. 2:**
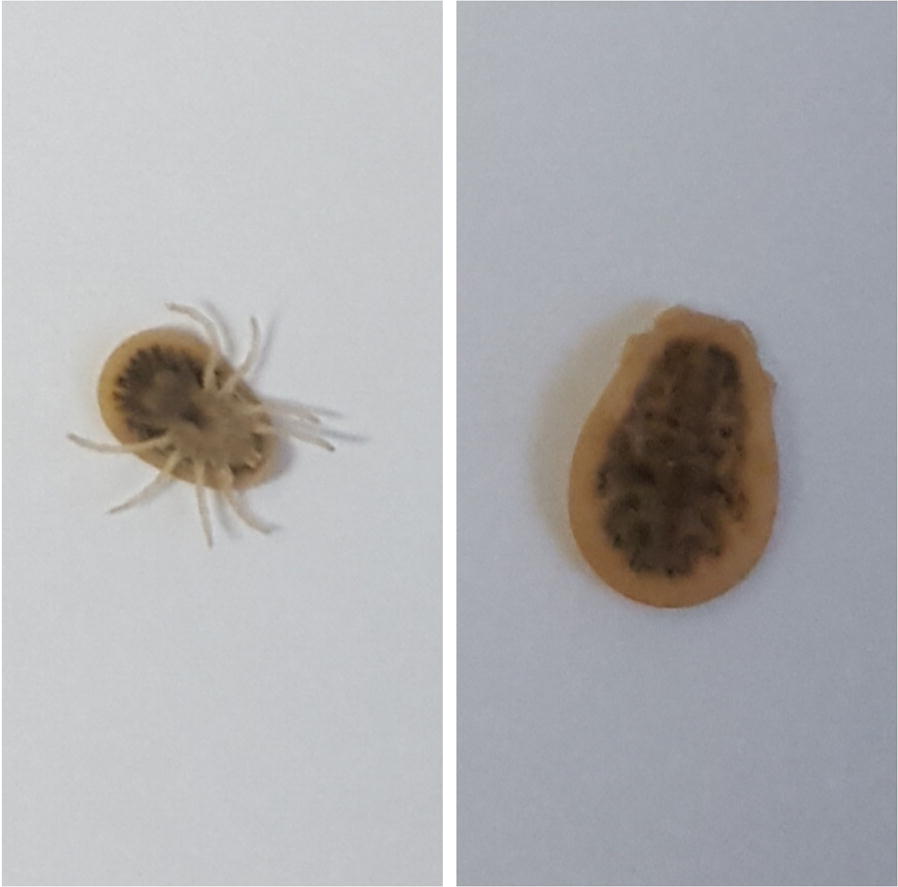
Specimens of *Argas reflexus*

After discharge, the patient referred to our Allergy Unit. An epinephrine autoinjector was provided and the patient was trained on its use. On the basis of clinical history, a reaction to drugs was excluded. In the day of the reaction, the patient referred she ate at dinner meat and vegetables, the same food she ate in the following days with no problems, before referring to allergy unit. Noteworthy, the patient reported a skin lesion of the left leg of about 15 centimetres of diameter which occurred 6 weeks before, characterized by local oedema and erythema, suggestive of insect or tick bite. The allergological work up included skin prick test and specific Immunoglobulins E (s-IgEs) to common foods and latex and prick + prick test with pork and beef kidneys and livers. On the basis of clinical history and negative skin test and s-IgEs, drugs, latex and foods containing alpha-gal were ruled out as causes of the reaction. Basal serum tryptase was in normal range (8.63 ng/ml). Total IgE level was 672 kU/l. Measurement of Immunoglobulin G and M class of *Borrelia burgdorferi* and *Rickettsia* antibodies was assessed with negative result. To explore the possibility of *A. reflexus* bite, we showed to the patient pictures of the tick and asked for the presence of pigeons in the domestic environment. Actually, the patient was able to collect in the immediate environment of the house a dozen of alive soft ticks, which were recognized as *A. reflexus* by an entomologist (Fig. 2).

## Discussion and conclusions

The present case report refers to a patient who experienced a near-fatal anaphylaxis with Kounis syndrome caused by *A. reflexus* bite. A suspected previous bite, which occurred 6 weeks before, characterized by a local skin lesion, possibly represented the primary sensitization. Concerning hymenoptera venom allergy, the risk of systemic reaction was reported to increase by 58% compared with controls if the first sting‐induced reaction has been preceded by a well‐tolerated sting within the 8 previous weeks [9]. We could speculate that a similar factor may elicit a systemic reaction to tick bite when a re-bite by *A. reflexus* occurs after a few weeks. As described above, the diagnosis was made only on the basis of history and clinical findings, because, though major allergen Arg r 1 has been isolated, allergen extracts for detection of s-IgEs are not commercially available for in vivo or in vitro tests. In conclusion, pigeon tick bites may explain cases of nocturnal anaphylaxis, otherwise generally diagnosed as idiopathic anaphylaxis [10]. Because of the growing number of pigeons in Middle and Southern Europe, *A. reflexus* should be taken into consideration by allergists as a cause of anaphylactic reactions.

## Data Availability

The datasets used during the current study are available from the corresponding author on reasonable request.

## Abbreviations

ECG: Electrocardiogram
s-IgEs: Specific immunoglobulins E
